# A novel shark single-domain antibody targeting OGT as a tool for detection and intracellular localization

**DOI:** 10.3389/fimmu.2023.1062656

**Published:** 2023-02-10

**Authors:** Xiaozhi Xi, Guokai Xiao, Guiqi An, Lin Liu, Xiaochun Liu, Peiyu Hao, Jennifer Yiyang Wang, Dandan Song, Wengong Yu, Yuchao Gu

**Affiliations:** ^1^ Key Laboratory of Marine Drugs, Ministry of Education, School of Medicine and Pharmacy, Ocean University of China, Qingdao, China; ^2^ Laboratory for Marine Drugs and Bioproducts of Pilot National Laboratory for Marine Science and Technology (Qingdao), Qingdao, China; ^3^ Key Laboratory of Glycoscience & Glycotechnology of Shandong Province, Ocean University of China, Qingdao, China; ^4^ College of Letters and Science Dept. of Microbiology, University of California, Los Angeles, Los Angeles, CA, United States

**Keywords:** O-GlcNAc, OGT, Shark VNAR, ELISA, immunofluorescence, computer simulation

## Abstract

**Introduction:**

O-GlcNAcylation is a type of reversible post-translational modification on Ser/Thr residues of intracellular proteins in eukaryotic cells, which is generated by the sole O-GlcNAc transferase (OGT) and removed by O-GlcNAcase (OGA). Thousands of proteins, that are involved in various physiological and pathological processes, have been found to be O-GlcNAcylated. However, due to the lack of favorable tools, studies of the O-GlcNAcylation and OGT were impeded. Immunoglobulin new antigen receptor (IgNAR) derived from shark is attractive to research tools, diagnosis and therapeutics. The variable domain of IgNARs (VNARs) have several advantages, such as small size, good stability, low-cost manufacture, and peculiar paratope structure.

**Methods:**

We obtained shark single domain antibodies targeting OGT by shark immunization, phage display library construction and panning. ELISA and BIACORE were used to assess the affinity of the antibodies to the antigen and three shark single-domain antibodies with high affinity were successfully screened. The three antibodies were assessed for intracellular function by flow cytometry and immunofluorescence co-localization.

**Results:**

In this study, three anti-OGT VNARs (2D9, 3F7 and 4G2) were obtained by phage display panning. The affinity values were measured using surface plasmon resonance (SPR) that 2D9, 3F7 and 4G2 bound to OGT with KD values of 35.5 nM, 53.4 nM and 89.7 nM, respectively. Then, the VNARs were biotinylated and used for the detection and localization of OGT by ELISA, flow cytometry and immunofluorescence. 2D9, 3F7 and 4G2 were exhibited the EC50 values of 102.1 nM, 40.75 nM and 120.7 nM respectively. VNAR 3F7 was predicted to bind the amino acid residues of Ser375, Phe377, Cys379 and Tyr 380 on OGT.

**Discussion:**

Our results show that shark single-domain antibodies targeting OGT can be used for in vitro detection and intracellular co-localization of OGT, providing a powerful tool for the study of OGT and O-GlcNAcylation.

## 1 Introduction

O-GlcNAcylation, an abundant protein post-translational modification, modifies thousands of nucleocytoplasmic proteins (1–4). These O-GlcNAcylated proteins are involved in many important biological processes, including the regulation of metabolism, proteasomal degradation, DNA replication and signal transduction (5). Aberrant O-GlcNAcylation is closely associated with the development of various diseases such as immune system disorders (6), cancer (7) cardiovascular disease (8) and diabetes (9). OGT attaches GlcNAc from glycosyl donors to serine/threonine residues of proteins. It is an important enzyme required for O-GlcNAcylation to occur. The regulation of OGT function is a hot topic of research in the fields of biology, biochemistry, medicine and pharmacology (10, 11). However, one of the main obstacles for the study of O-GlcNAcylation and OGT is the lack of favorable research tools.

Antibodies with only heavy chains (HCAbs) were first reported by researchers from camelids in the early 1990s (12). Two years later, it was discovered that sharks also possessed a type of antibody with only two heavy chains, termed immunoglobulin neoantigen receptors (IgNARs) (13). Variable structural domains (VNARs) of IgNARs identify naturally occurring independent heavy chain-only binding structural domains with a molecular weight of ~12 kDa (14). VNARs differ from classical and single-domain camelid antibodies due to the lack of complementarity determining region 2 (CDR2) (15, 16). In addition, VNARs have two highly variable loops (HV2 and HV4) (17, 18). VNARs have a longer CDR3 segment than classical antibodies and form an additional intercellular disulfide bond, allowing for more stable structure from VNARs while recognizing invisible epitopes on target antigens (19, 20). Furthermore, VNARs can be produced at low cost by non-mammalian expression systems (21) and exhibit good stability under a variety of conditions (22). In addition to these unique features, due to the small size of VNARs, they have strong potential for applications in super-resolution imaging (23).

Here, two *whitespotted bambooshark* were immunized with recombinant OGT protein. Then, an anti-OGT VNAR phage display library was constructed using mRNA from the immunized shark peripheral blood lymphocytes (PBLs). After three panning cycles, three VNARs targeting OGT were isolated. The three VNARs of 2D9, 3F7 and 4G2 were expressed in *E. coli*. The affinities of the three VNARs were examined. The intact molecular weight of recombinant VNARs was assayed by LC-MS/MS. The VNARs can be used for ELISA and immunofluorescence assays. VNAR 3F7 was predicted to bind with OGT *via* amino acid sites of Ser375, Phe377, Cys379 and Tyr 380. This study provides a new tool for the research of OGT and O-GlcNAcylation.

## 2 Methods and materials

### 2.1 Expression and purification of OGT recombinant protein

The recombinant plasmid pET-28a-OGT was preserved by our laboratory. The expression and purification experiments were carried out as descripted previously (24). Briefly, the plasmid DNA was transformed into *E. coli* BL21 (DE3) cells and induced with isopropyl-β-D-thiogalactoside (IPTG). The induced bacterial was collected by centrifugation and fragmented. Ni-NTA column (GE Healthcare 17-5268-02, USA) was used to purify the recombinant OGT protein. OGT expression and purification were assessed by sodium dodecyl sulfate polyacrylamide gel electrophoresis (SDS-PAGE).

### 2.2 Shark immunization

Two male *Chiloscyllium plagiosum* sharks were first immunized with recombinant ncOGT 100 µg emulsified in complete Freund’s adjuvant (Sigma-Aldrich, USA). Two weeks later, the sharks were immunized by emulsifying 12 µg of ncOGT using incomplete Freund’s adjuvant. Immunization was carried out every fortnight. The final booster was administered with 2 µg ncOGT dissolved in phosphate-buffered saline (PBS) through intravenous injection. Blood samples were collected at week 0 (before immunobleeding), weeks 6, 8 and 10. PBLs were isolated and total RNA was prepared as described by Vincke et al. (25).

### 2.3 Detection of OGT specific IgNAR in shark serum

The titers of shark serum IgNAR were measured using ELISA. Immunized shark plasma was diluted in a 4% (w/v) milk PBS (MPBS) gradient (1:10; 1:100; 1:1000; 1:10,000; 1:100,000) and added to ELISA plates. Detection was carried out using a laboratory-prepared rabbit monoclonal antibody against shark (26). Then, a 1:5000 dilution of anti-rabbit IgG-HRP antibody was added to the plates and incubated at 37°C for 1 h. Finally, TMB colour solution was added to each well and incubated at 37°C for 15 minutes. The reaction was stopped with 1 M H_2_SO_4_ and the optical density (OD) 450 was measured.

### 2.4 Phage display library construction

Total RNA was extracted from the shark’s PBLs and used as templates to synthesizing first-strand cDNA using oligo(dT) primers. The library encoding sequences were amplified by PCR from cDNA, the framework specific primers Bam VF1 CGCGGCCCAGCCGGCCATGGCCGCCSMACGGSTTGAACAAACACC and Bam VF2 CGCGGCCCAGCCGGCCATGGCCGCCGCACGGGTTGAACAAACACCG. DNA fragments were cleaved with restriction enzymes *Nco* I and *Not* I (NEB) for use in subsequent experiments. An anti-OGT phage display library of about 10^8^ independent transformants was obtained following the detailed protocol as Ubah et al. (27).

### 2.5 Selection from libraries by phage display

Library amplification was carried out overnight at 30°C in 2 x TY medium, ampicillin and kanamycin. The phage was precipitated and pannning. For the first round of panning, immunotubes were washed 10 times with PBS containing 0.1% Tween 20 (PBST). For the second and third rounds, immunotubes were washed 20 times with PBST at the time of panning. After the third round of panning, the screened phages were detected by polyclonal and monoclonal phage ELISA and the results were used to evaluate the effectiveness of the panning. the ELISA method was similar to that in 2.4, with the difference that the HRP-conjugated anti-M13 antibody (1:1000) was used as a secondary antibody and OD 450 was measured using a microplate reader.

### 2.6 Shark VNAR protein expression and purification

The VNAR gene was amplified by PCR and cloned into pET-28a by *Nco* I and *Not* I restriction sites. The recombinant plasmid was identified by sequencing. The expression and purification method was similar to the OGT preparation method in 2.1.

### 2.7 VNAR affinity determination

Biomolecular interaction analysis (BIA) was used to analyse the affinity of shark single-domain antibodies (28).. Anti-OGT VNARs were diluted to a concentration of 100 nM. Recombinant ncOGT protein was diluted to a series of different concentrations with PBS: 50, 100, 200, 400 and 800 nM; Calibration solution (PBST solution containing 1% glycerol) and OGT solution with gradient dilution were introduced successively. The binding time was 120 s. PBS buffer was introduced, and the dissociation time was 200 s. After dissociation, phosphoric acid (1:100 dilution) regeneration solution was introduced, and the regeneration time was 200 s. The response data were normalized using Langmuir combined with model fit analysis (Biacore evaluation software).

### 2.8 Molecular weight analysis

Antibody samples are processed by adding 200 μL ddH2O to a 10 kDa ultrafiltration tube, 11000 g, centrifuged for 1 min, and removing a small amount of glycerol from the ultrafiltration tube. Add 200 μg of antibody to the ultrafiltration tube, 11000 g for 3 min. Add 200 μL of ddH2O to displace the sample once for desalination. UPLC separation was performed using an ACQUITY UPLC Protein BEH C4 Column, 300 Å, 1.7 µm, 2.1 mm x 50 mm. The column temperature was set at 80°C and UPLC separation was performed using solvent A (0.1% v/v FA in water) and solvent B (0.1% FA in acetonitrile). The flow rate of the UPLC was 0.3 ml/min and the mobile phase B was eluted from 10% to 95% in 8 min using a gradient elution. LC-MS TIC (total ion count) data were collected in the resolution mode in the m/z range 500-3,500. The mass spectrometry raw data were processed using BiopharmaLynx software (v 1.2) in complete protein mode at a resolution of 10,000, mass matching tolerance was set at 20 ppm.

### 2.9 Biotin labeling of single-domain antibody and establishment of the indirect ELISA

Anti-OGT VNARs were dissolved 1 mg/mL with PBS. 10 mM solution of biotin reagent was immediately prepared in an organic solvent dimethyl sulfoxide (DMSO). The protein solution was spiked with the appropriate amount of 10 mM NHS-LC-Biotin reagent (Cat. No.: C100212/C100215, angon Biotech) and the reaction was incubated on ice for 2 h. At this point the labelling of the protein had been completed. Although, there was still excess un-reacted and un-hydrolyzed biotin reagent in the solution, the labelled protein could usually be tested initially by means of a biotin quantification kit (29).

OGT proteins (1, 2, 4 and 10 μg/mL) were directly immobilized on 96-well plates at 4°C and the optimal coating antigen concentration (2667-0.2667 nM) of biotinylated anti-OGT VNARs were determined by indirect ELISA. ELISA plates were closed with 3% BSA in PBST. Follow-up was similar to project 2.4, with the difference that binding of anti-OGT VNAR was shown with HRP-conjugated streptavidin (1:5000) secondary antibody. At the end of the reaction, absorbance was measured at 450 nm.

### 2.10 Western blot

NCI-H1299 cells were lysed in SDS lysis buffer (1% SDS, 50 mM Tris-HCl pH 7.5, 100 mM NaCl, and Complete™ Protease Inhibitor). Lysates were resolved on 4-12% SDS polyacrylamide gels (SDS PAGE), transferred to Immobilon-FL PVDF membranes (#IPVH00010, MerckMillipore), and immunoblotted with the indicated antibodies. Blots are identified with the antibody, dilution and clone/catalogue number in parentheses. The antibodies used were anti-OGT (1:1000, ab96718, Abcam), Goat Anti-Rabbit IgG H&L (HRP) (1:2000, ab6721, Abcam), and HRP-Streptavidin (1:5000, RABHRP3, Sigma-Aldrich).

### 2.11 Flow cytometry

Wild-type NCI-H1299 cells and OGT-silenced NCI-H1299 (lab constructs for preservation) (30) were collected, washed with PBS, and then fixed and permeabilized with 4% paraformaldehyde and 0.2% Triton-100. Cells were incubated with FACS buffer (1 x PBS with 0.5% BSA) containing 10 µg/ml of commercial OGT antibody (ab96718, Abcam), 2D9 and 3F7 for 30 min at 4°C. Subsequently, cells were washed twice with FACS buffer and stained with goat anti-rabbit (ab150077, Abcam) coupled to A488 and anti-His fluorescent antibody (EPR20547, Abcam). Cells were washed twice with PBS. Samples were analyzed by flow cytometry.

### 2.12 Transfections

NCI-H1299 cells were seeded on glass coverslips of six-well plates at 1 x 10^5^ cells per well. After cell apposition, the p3×Flag-OGT plasmid was transfected into the cells using Attractene Transfection reagents (QIAGEN), according to the manufacturer’s instructions.

### 2.13 Immunofluorescence and co-localization

NCI-H1299 cells were seeded at 5 x 10^4^ cells per well on glass slides and incubated for 16 h. Fixation and permeabilization were performed with 4% paraformaldehyde and 0.1% Triton X-100. Cells were incubated overnight at 4°C with anti-OGT/O-linked N-acetylglucosaminyl transferase antibody (ab96718) and biotinylated anti-OGT VNAR (1 μg/ml). Detection of antigen-bound VNARs was achieved by the addition of anti-6-His A488 MAbs (CST, #14930). Nuclear staining was then performed with 4’,6-diamidino-2-phenylindole (DAPI). Images were obtained using fluorescence microscopy.

NCI-H1299 cells transfected with p3×Flag-OGT plasmid were spread on glass coverslips in 24-well plates grown overnight and then subjected to immunofluorescence co-localization. An Alexa Fluor^®^ 488 fluorescent Anti-DDDK tag (ab205606) was used to identify the OGT within the transfected cells. Antibody detection was similar to the immunofluorescence operation.

### 2.14 Computational modeling of OGT proteins and VNAR

The 3D structures of single-domain antibodies and ncOGT were predicted by SWISS MODEL (31), and the top ranking was selected as their 3D structure. VNARs (PDB ID: 7FBK (32)) and OGT (PDB ID: 7NTF (32) were predicted to be suitable 3D structures. ZDock (33) and PDBePISA (34) were used to obtain binding patterns between VNARs and OGT. First, ZDock was used to initially explore the location of VNAR and OGT, and 10 predictive composite models with good fit were screened. PDBePISA was used to identify the binding chains and binding sites of OGT and 3F7. The binding chains for these two protein interactions can be obtained from the analysis.

### 2.15 Statistical analysis

The data were analyzed using Graph Pad Prism (version 5.0), three times in parallel for each set of experiments. All raw MS data were analyzed using the MNIFI software (Waters Company, U.K.). Data were presented as the mean ± standard deviation.

## 3 Results

### 3.1 Recombinant purification of OGT and animal immunization

**Figure 1:**
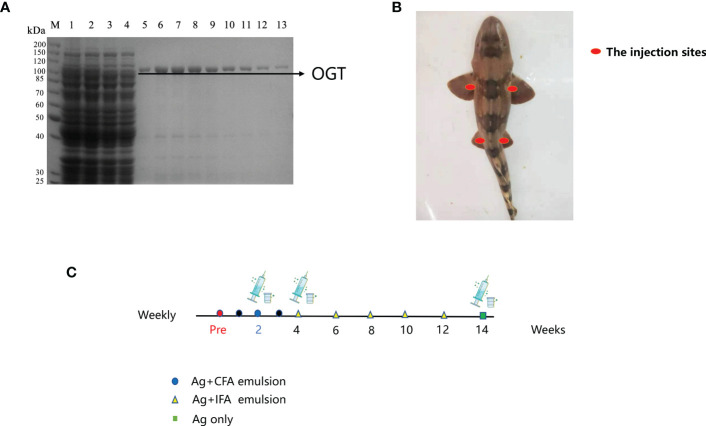
Immunization of bamboo sharks with OGT. **(A)** Results of ncOGT protein isolation and purification. MW, protein molecular markers; Lane 1: bacterial lysate supernatant by pET28a-ncOGT; Lane 2: penetrating liquid; Lane 3: 20 mM imidazole eluent; Lane 5-13: the purified OGT protein. SDS-PAGE gel was stained using Coomassie Brilliant Blue. **(B)** The injection and bleeding sites. **(C)** The immunization schedules.

The OGT was expressed and purified by the *E. coli* expression system. SDS-PAGE analysis was performed and no major impurities from *E. coli* were seen (**Figure 1A**). A good quality antigen was produced and used to immunize shark. To promote effective immunity in sharks, we optimized immunization parameters, including route of administration and injection site (**Figures 1B, C**). Shark antigen-driven immune responses were determined by measuring IgNAR titers in serum (pre- and post-bleeds, respectively). An increase in OGT-specific IgNAR titers were observed after successive immunization boost up.

### 3.2 The Construction of shark VNAR phage display immune library

**Figure 2:**
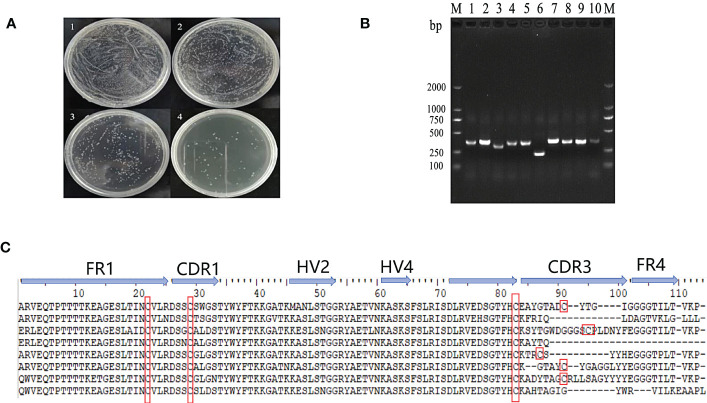
Construction and quality evaluation of phage library. **(A)** Determination of the titer of the single domain antibody phage library. 1-4: The dilution of original bacterial solution for 10^-2^, 10^-3^, 10^-4^ and 10^-5^ times respectively. **(B)** Detection of positive rate of single domain antibody library phage by PCR; **(C)** Sequences of VNAR domains from the randomly selected ten colonies of the phage library. FR is framework region; CDR is complementarity-determining region; HV is hypervariable region. Canonical Cys residues are enclosed in red.

The VNAR sequences were amplified from isolated PBLs by PCR and cloned into a phage vector containing the M13 phage truncated coat protein PIII gene in frame. The display library size was estimated to be 2.65 x 10^8^ transformants (**Figures 2A, B**). Ten single clones of the library formed by the construct were randomly selected for sequencing to determine the quality of the library. The results showed that 90% of the libraries incorporated VNAR sequences and 80% encoded functional inserts with a unique amino acid sequence in CDR3 (**Figure 2C**).

### 3.3 Isolation of Anti-OGT VNARs by phage display

**Figure 3:**
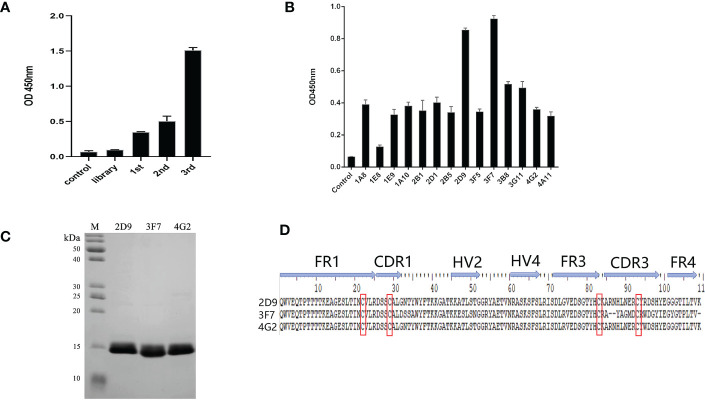
Selection of anti-OGT VNARs. **(A)** Detecting the titer of phage single domain antibody library in each round of panning; **(B)** Detection of specific bacteriophage by monoclonal ELISA; **(C)** Characterization and analysis of molecular weight of 2D9,3F7 and 4G2; **(D)** Amino acid sequence alignment of the three anti-OGT VNARs. FR is framework region; CDR is complementarity-determining region; HV is hypervariable region. Canonical Cys residues are enclosed in red.

OGT-specific shark VNARs were screened out by three rounds of phage panning with decreasing OGT concentration. After three rounds of panning, the specific OGT phage was enriched (**Figure 3A**). Fourteen clones from the third panning of the phage library were selected by monoclonal phage ELISA (**Figure 3B**). Three VNARs, named as D9, 3F7 and 4G2, were identified by sequencing, and they were efficiently expressed in *E. coli* and purified (**Figure 3C**). By analyzing the sequences of the three VNARs, we found that the VNARs belong to type II VNAR. In type II VNAR, a cysteine residue was found in FR1, FER3, CDR1 and CDR3, respectively, a result consistent with those reported in the literature (**Figure 3D**) (19).

### 3.4 Affinity kinetics of Anti-OGT VNARs

**Figure 4:**
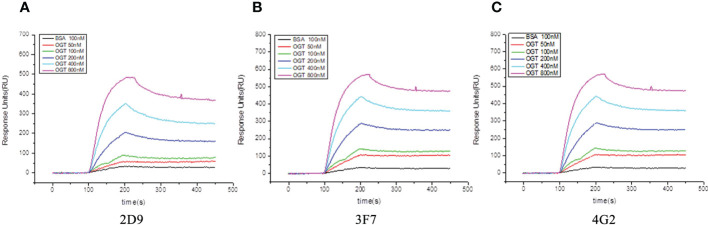
Sensorgram of biomolecular interaction analysis of VNARs binding to OGT. The binding activity of anti-OGT VNARs was analyzed at concentrations between 50 nM and 800 nM. **(A)** 2D9; **(B)** 3F7; **(C)** 4G2.

Biacore was used to detect the affinity of the three anti-OGT VNARs. The detected affinity determination results and kinetic parameters are shown in **Figure 4** and **Table 1**. The equilibrium dissociation constants (K_D_) of three single domain antibodies combined with ncOGT are 3.55, 5.34 and 8.97×10^-8^ M respectively. The results showed that the three VNARs, especially 2D9 and 3F7, bind OGT with good affinity (**Figures 4A, B, C**).

**Table 1:** The affinity kinetics analysis of anti-OGT VNARs by SPR.

VNAR	kon (M^-1^S^-1^)	kdis (S^-1^)	K_D_ (M)
2D9	1.9×10^-4^	6.75×10^-4^	3.55×10^-8^
3F7	1.98×10^-4^	1.06×10^-3^	5.34×10^-8^
4G2	2.56×10^-4^	7.6×10^-4^	8.97×10^-8^

### 3.5 Molecular weight analysis of Anti-OGT VNARs

**Figure 5:**
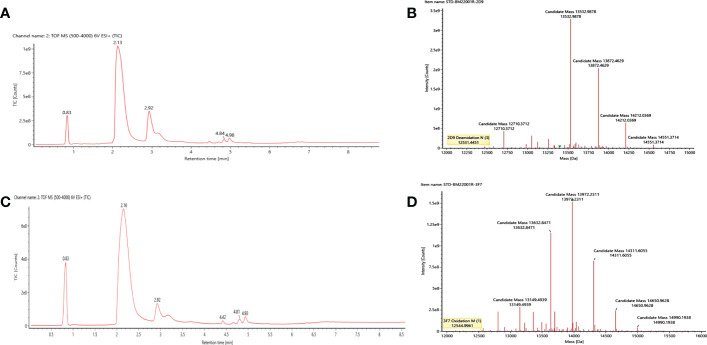
Molecular weight determination results of the intact anti-OGT VNARs. **(A)** Original mass spectrum of the intact 2D9. **(B)** Deconvolution diagram of the intact 2D9. **(C)** Original mass spectrum of the intact 3F7. **(D)** Deconvolution diagram of the intact 3F7.

In order to efficiently characterize and qualify the VNARs, the complete molecular weight of 2D9 and 3F7 were determined by LC-MS/MS. LC-MS/MS data were analyzed using BioFinder 3.0 software. The total ion chromatogram was shown in **Figures 5A, C**, and deconvolution plots were shown in **Figures 5B, D**. The molecular masses of VNARs were indicated in **Table 2**. The relative molecular mass of the primary peak of 3F7 and 2D9 were 12544.9961 and 12531.4451 respectively, which deviated from the theoretical relative molecular mass by 1.18 and 0.674, which was within the error range.

**Table 2:** Relative molecular mass analysis results for anti-OGT VNARs.

Protein name	Response	Observed mass (Da)	Expected mass (Da)	Mass error (mDa)	Mass error (ppm)
2D9	2973783	12531.4451	12530.7706	674.5	53.8
3F7	2185044	12544.9961	12543.8157	1180.4	94.1

### 3.6 Generation and determination of biotinylated Anti-OGT VNARs

**Figure 6:**
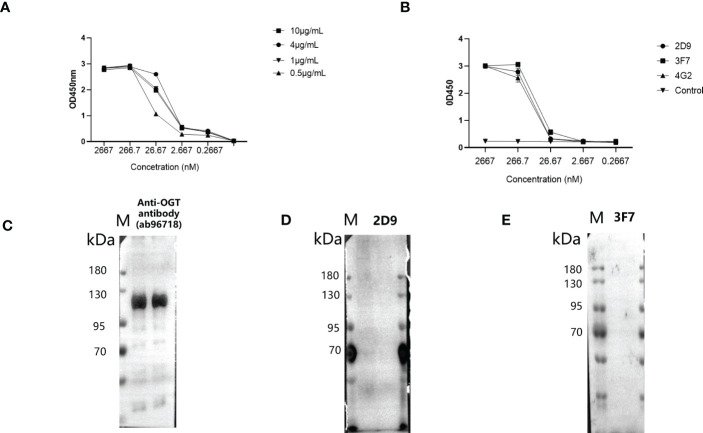
Evaluation of specificity and reactivity of the biotinylated anti-OGT VNAR. **(A)** Determination of the optimal conditions of antigen. **(B)** The reactivity of VNAR against OGT was detected by indirect ELISA. C-E. NCI-H1299 whole-cell extracts were analyzed by WB using the commercial anti-OGT antibody **(C)**, 2D9 **(D)** and 3F7 **(E)**, respectively.

To facilitate the application, they were labelled with biotin. Biotinylated VNARs were prepared and used to detect OGT protein by ELISA. The results show that optimal concentration of antigen for OGT protein was 1 μg/mL (**Figure 6A**). As shown in **Figure 6B**, the VNARs were exhibited high reactivity, and the EC_50_ values of the biotinylated 2D9, 3F7 and 4G2 were identified as 102.1, 40.75 and 120.7 nM by indirect ELISA. Combined with the above results, 3F7 was shown to be the best one (**Figure 6B**). To investigate whether the anti-OGT-VNARs can be used for Western blot (WB) analysis, NCI-H1299 cell lysates were used for the detection. The commercial anti-OGT antibody (as a positive control) can detect the OGT protein expressed in NCI-H1299 cells (**Figure 6C**). However, there was no band when NCI-H1299 cell lysates were examined by WB using 2D9 and 3F7 (**Figures 6D, E**). The reasons might be that the antigen used in immunizing the sharks and panning is native recombinant OGT protein, which produces antibodies that may only bind conformational epitopes, whereas WB experiments need antibodies that can bind linear epitopes of the antigen. Therefore, anti-OGT VNARs prepared in this study cannot be used in WB analysis.

### 3.7 Binding analysis of Anti-OGT VNARs to cells

**Figure 7:**
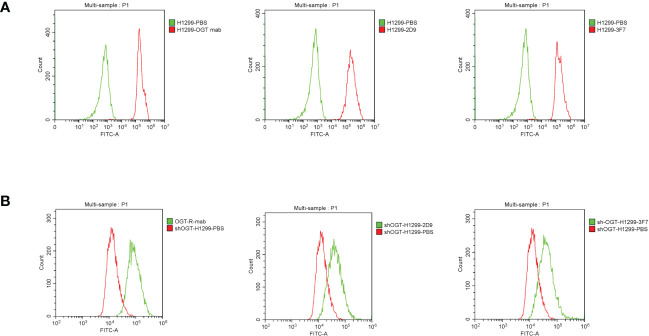
Flow cytometric evaluation of the binding ability of shark nanobodies to cells. **(A)** and **(B)** Binding of commercial anti-OGT monoclonal antibodies, shark single domain antibodies 2D9 and 3F7, to wild type H1299 cells **(A)** and OGT-silenced NCI-H1299 **(B)**.

We examined the ability of the antibody to bind to the cells using flow cytometry. In our experiments, we used NCI-H1299 cells previously constructed in the laboratory with OGT-silenced NCI-H1299 and wild type NCI-H1299 cells. The results showed that our prepared shark nanobodies 2D9 and 3F7 could bind to NCI-H1299 cells highly expressing OGT and the binding effect was comparable to that of commercial OGT (**Figure 7A**). At the same time, the binding of anti-OGT antibodies decreased in the OGT-silenced NCI-H1299 (**Figure 7B**), demonstrating that the antibodies have specific recognition of intracellular OGT.

### 3.8 Immunofluorescence analysis of intracellular OGT by VNARs

**Figure 8:**
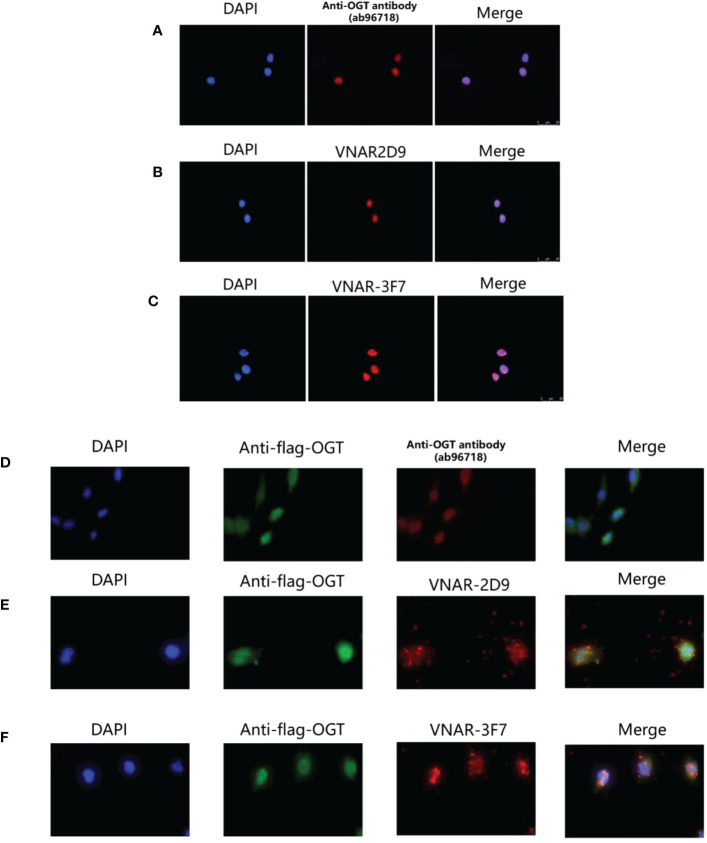
Cytosolic and cytoplasmic localization of OGT. **(A)** Binding of anti-OGT/O-linked N-acetylglucosaminyltransferase (ab96718); **(B)** 2D9; **(C)** 3F7 in wild-type H1299 cells, respectively. Immunofluorescence images representing OGT (Red) and DAPI (Blue) were overlaid (Merge) to show localization within the nucleus. **(D–F)**. Commercial anti-OGT antibody **(D)**, 2D9 **(E)**, and 3F7 **(F)** were each co-localized with p3×flag-ncOGT transfected H1299 cells. Immunofluorescence images of OGT represented in green, the three antibodies represented in Red and DAPI Blue were overlaid (Merge) to show co-localization.

To assess the function of OGT VNARs, 2D9 and 3F7 were used to detect the localization of OGT in cells by immunofluorescence microscopy. It had been reported in some literature that OGT proteins were localized in the nucleus along cytoplasmic (35). We localized OGT using commercial antibody (**Figure 8A**), 2D9 (**Figure 8B**) and 3F7 (**Figure 8C**) by immunofluorescence, the results showed that most of OGT (Red) localized in nucleus (Blue) in NCI-H1299 cells as previously reported (36). To further confirm the specific binding of VNARs to OGT, we transfected Flag-tagged OGT in NCI-H1299 cells for immunofluorescence analysis, as shown in **Figures 8D–F**. Anti-Flag antibody (Green) and VNARs (Red) had strong co-localization in the nucleus and cytoplasm of NCI-H1299 cells, as can be seen in the Merge section. These results suggest that anti-OGT VNARs could be suitable candidate tools for intracellular imaging.

### 3.9 Models of VNARs-OGT complexes

**Figure 9:**
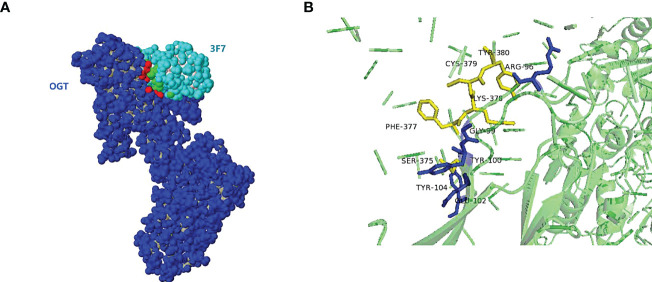
The 3D model for 3F7-OGT. **(A)** Surface representation of the VNAR 3F7 and OGT. OGT (blue) and 3F7 (green). **(B)** Cartoon representation of VNAR 3F7-OGT model. VNAR 3F7 hydrogen bond sites (Blue stick) and OGT (Yellow sticks). The residue ARG96/GLY99/TYR100/GLU102/TYR104 in VNAR 3F7 establishing hydrogen bond with a conserved SER375/PHE377/CYS379/TYR380 in the OGT receptors.

To predict the binding site of OGT and VNAR 3F7, the 3D model for 3F7-OGT complex was generated by merging the homology models developed for 3F7 and OGT. Complex models of 3F7 and OGT were constructed using ZDOCK server based on VNAR 3F7 (PDB ID 7BFK) and OGT crystal structures (PDB ID 7NTF) (**Figure 9A**), respectively. PDBePISA was applied for identification of possible binding sites. The residue ARG96/GLY99/TYR100/GLU102/TYR104 in VNAR 3F7 QA74: Table/Figure XXX has not been mentioned in the article. Please add a citation within the text, noting that Figures and Tables must appear in sequence.establishing hydrogen bond with a conserved SER375/PHE377/CYS379/TYR380 in the OGT receptors was formed in the generated model signifying its validity (**Figure 9B**).

The TPR repeat consists of 34 highly conserved amino acid residues, which are mainly involved in the binding of OGT to substrate proteins (37). The grooves in the supercoiled structure on the right contain amino acid residues involved in binding the target proteins of OGT (38), and the protein-binding ability of OGT is affected by the number of TPRs (39). The predicted four amino acid binding sites of OGT were located on TPR13, which is shared by all the three types of OGT isoforms ncOGT, mOGT and sOGT, indicating that VNAR 3F7 can facilitate the in-depth research on the functional of OGT.

## 4 Discussion

O-GlcNAc is a universal protein modification with a variety of important physiological functions. The addition of GlcNAc to protein is catalyzed by a unique OGT. The study of protein O-GlcNAc function has been limited by the lack of suitable research tools and methods (40, 41). Therefore, in order to further explore the physiological and pathological role of OGT, high specificity antibodies against OGT remain to be prepared. The structure in VNARs and the characteristics of the composition of the individual Loop loops allow them to recognize more hidden epitopes, and therefore VNARs may play an unexpected role in development studies of OGT and O-GlcNAcylation. Conventional monoclonal antibodies are limited in their in-depth application by their large size, complex structure and sensitivity to extreme environmental temperatures (42). Shark VNAR is better able to penetrate tissues and dense structures and penetrate deeper into the target protein.

In this study, three shark VNARs (2D9, 3F7 and 4G2) against OGT were screened by phage display assay. The three single-domain antibodies were purified to obtain high purity and high affinity antibodies. Their K_D_ values could reach above 10^-8^ M, and it is well documented that K_D_ values below 10^-8^ M indicate a high affinity between the antigen and the antibody (14, 16, 21). Shark VNARs were successfully prepared against OGT and these were then labelled with biotinylated markers for subsequent antibody application. Chemical biotinylation is the binding of biotin to a non-specific covalent bond in the target molecule thereby linking the two (43). In our experiment, chemical biotinylating was used. Polyclonal or monoclonal antibodies were often used in traditional ELISA methods, but the quality of polyclonal antibodies could be inconsistent from batch to batch and the process of industrial mass production of monoclonal antibodies can be complex. VNARs could be easily produced in recombinant protein expression systems with hosts including bacteria and yeast. This advantage allowed VNARs to be produced on a large scale and ensures batch-to-batch consistency. Additionally, single domain antibody can be intracellular expressed, which can be used for live-cell imaging. Then, in order to confirm the application scope of the antibody. The nucleus localization of OGT was verified by immunofluorescence, suggesting that anti-OGT VNARs might be used as a powerful tool for super-resolution imaging of OGT. The amino acid binding site of 3F7 to OGT was analyzed using computer simulations. The protein-binding site of OGT is a discontinuous epitopes located on TPR13 domain, which is consistent with the results that 3F7 can’t be used for WB assay.

## 5 Conclusion

In this study, three VNARs against OGT proteins were isolated from an immune phage display VNAR library. VNAR 3F7 was shown to be more reactive, sensitive and reproducible. Immunofluorescence and ELISA have shown that OGT can be accurately detected by this VNAR with results comparable to those of commercial antibodies. Therefore, this small single domain antibody would contribute to the research of OGT and O-GlcNAcylation *in vitro*. This VNAR would like to have further applications for live-cell imaging of OGT by VNAR intracellular expression and super-resolution fluorescence imaging.

## Data availability statement

The GenBank Submissions Staff and have received confirmation of the email address. Data already available on NCBI.2D9: https://www.ncbi.nlm.nih.gov/search/all/?term=OQ065564. 3F7: https://www.ncbi.nlm.nih.gov/search/all/?term=+OQ065565. 4G2: https://www.ncbi.nlm.nih.gov/search/all/?term=+OQ065566.

## Ethics statement

The animal study was reviewed and approved by Institutional Animal Care and Use Committee of Ocean University of China.

## Author contributions

YG proposed the concept and design of the study. XX and GX wrote the article. XXi, GX and DS conducted the research and validation of the experiment. XXand GX constructed the graphs and tables. LL, XL, GA, JW and PH collected and analyzed the data. YG, WY and XX were responsible for the accuracy of the study and the review of the article. All authors contributed to the article and approved the submitted version
